# Anti-phosphatidylserine antibody levels are low in multigravid pregnant women in a malaria-endemic area in Nigeria, and do not correlate with anti-VAR2CSA antibodies

**DOI:** 10.3389/fcimb.2023.1130186

**Published:** 2023-03-28

**Authors:** Adebimpe Fasanya, Nurat Mohammed, Bandar Hasan Saleh, Muyideen Kolapo Tijani, Alexandra Teleka, Maria del Pilar Quintana, Lars Hviid, Kristina E. M. Persson

**Affiliations:** ^1^ Cellular Parasitology Programme, Cell Biology and Genetics Unit, Department of Zoology, University of Ibadan, Ibadan, Nigeria; ^2^ Department of Laboratory Medicine, Division of Clinical Chemistry and Pharmacology, Skåne University Hospital, Lund University, Lund, Sweden; ^3^ Department of Medical Microbiology and Parasitology, King Abdulaziz University, Jeddah, Saudi Arabia; ^4^ Department of Immunology and Microbiology, Faculty of Health and Medical Sciences, University of Copenhagen, Copenhagen, Denmark; ^5^ Department of Infectious Diseases, Rigshospitalet, Copenhagen, Denmark

**Keywords:** malaria, phosphatidylserine, anemia, gravidity, antibody, VAR2CSA, *P. falciparum*

## Abstract

Anemia is a common malaria-associated complication in pregnant women in endemic regions. Phosphatidylserine (PS) is exposed to the immune system during the massive destruction of red blood cells (RBCs) that accompany malaria, and antibodies against PS have been linked to anemia through destruction of uninfected RBCs. We determined levels of anti-PS IgG antibodies in pregnant women in Ibadan, Nigeria and correlated them to parameters of importance in development of anemia and immunity. Anti-PS correlated inversely with Packed Cell Volume (PCV), indicating that the antibodies could contribute to anemia. There was no correlation with anti-VAR2CSA IgG, haptoglobin or parasitemia, indicating that the modulation of anti-PS response is multifactorial in nature. Anti-PS levels were lowest in multigravidae compared to both primigravidae and secundigravidae and correlated inversely with age. In conclusion, lower levels of anti-PS in multigravidae could be beneficial in avoiding anemia.

## Introduction

Pregnant women and children disproportionately carry the burden of malaria in endemic areas (WHO, 2021). Anemia is central to the pathology of malaria in this vulnerable group, and contributing factors are dyserythropoesis and destruction of both infected and uninfected RBCs (White, 2018). Notably, about eight uninfected RBCs are estimated to be removed from the circulation for every *P. falciparum*-infected RBC destroyed (Jakeman et al., 1999; Price et al., 2001) and even more uninfected RBCs, about 34, are removed during *P. vivax* infection (Collins et al., 2003). Malarial anemia has also been associated with loss of complement regulatory proteins such as CR1 and CD55 in *P. vivax* and *P. falciparum* infections (Oyong et al., 2018). This loss of complement regulatory proteins has also been observed during uncomplicated cases of malaria (Oyong et al., 2019). Individuals living in endemic areas often experience post-treatment anemia even after complement regulatory protein levels, such as CD55 or CR1, have normalized (Stoute et al., 2003). This clearly shows that there are other unknown erythrocyte lysis mechanisms associated with malaria.

Alongside protective antibody responses against *P. falciparum* parasites (Tijani et al., 2021), auto-antibodies targeting DNA (Adebajo et al., 1993), erythrocyte membrane proteins and their associated glycan moieties (Berzins et al., 1983; Ravindran et al., 1988; Saleh et al., 2022) and phospholipids (Adebajo et al., 1993; Fernandez-Arias et al., 2016; Rivera-Correa et al., 2017) have been reported. Phosphatidylserine is a membrane inner leaflet phospholipid that has been shown to become exposed on *P.falciparum*-infected RBC membranes (Maguire et al., 1991; Sherman et al., 1997; Pattanapanyasat et al., 2010). The exteriorization of PS on infected cells, which is a hallmark of eryptosis, is induced by the activation of non-selective cation channels and the activation of scramblase by an influx of Ca^2+^ (Lang et al., 2004; Fraser et al., 2021). Interestingly, PS exteriorization has also been demonstrated in uninfected RBCs in *P.falciparum in vitro* cultures (Engelbrecht and Coetzer, 2016) and also in malaria murine models (Totino et al., 2010; Fernandez-Arias et al., 2016). Autoimmune antibody responses against membrane PS contribute to anemia in mice (Fernandez-Arias et al., 2016) and in naturally infected humans, where they correlated with low hemoglobin levels (Barber et al., 2019). Elevated PS antibodies were also found in children with severe malaria and correlated with markers of kidney damage (Rivera-Correa et al., 2019a). The mechanisms through which PS antibodies are produced during malaria are not completely understood, but atypical FcRL5^+^T-bet^+^ B cells have been shown to be their source in malaria-naïve individuals (Rivera-Correa et al., 2019b).

Placental malaria contributes significantly to maternal anemia and to low birthweight and neonatal mortality (Brabin et al., 2004; Rogerson et al., 2007). During pregnancy, *P. falciparum*-infected RBCs express VAR2CSA and sequester in the placenta. VAR2CSA is a 350 KDa protein that is composed of six Duffy-binding-like domains and mediates infected RBC sequestration by binding to oncofetal chondroitin sulphate, which is exclusively expressed by the syncytiotrophoblasts in the placenta (Fried and Duffy, 1996; Salanti et al., 2004; Salanti et al., 2015). Antibodies against VAR2CSA increase in naturally exposed pregnant women in a parity-dependent manner and their elevated levels have been associated with protection from anemia, low-birth weight, and placental infection (Duffy and Fried, 2003; Staalsoe et al., 2004; Feng et al., 2009). Furthermore, measurement of anti-VAR2CSA antibodies is a useful serosurveillance tool that can be used in endemic regions due to the association with *P. falciparum* transmission across different geographic zones (Fonseca et al., 2019). Understanding antibody responses to VAR2CSA is complicated by its relatively large size and multi-domain structure, but antibodies to full-length VAR2CSA have been found to be more predictive of placental infection than antibodies against the DBL single domains (Cutts et al., 2020). By using VAR2CSA-specific antibodies as a marker of exposure, the current study was carried out to determine the relationship between anti-PS antibodies and level of placental malaria exposure of pregnant women living in Ibadan, an endemic area located in the southwest of Nigeria.

## Materials and methods

### Ethical consideration

Ethical approvals for this study were obtained from the Oyo State Ministry of Health Ethical Review Approval Committee (AD 13/479/833), the Ethical Committee Board of Our Lady of Apostle Hospital, Oluyoro, Okeofa, Ibadan (OCH/EC/18/08) and the Swedish Ethical Review Authority (2022-00777-01). Permission was also obtained from the administrative heads of each hospital where samples were collected. A signed consent form was obtained from each participant before samples were collected. Study participation was voluntary and refusal did not attract any penalty with regards to the benefits of the study. The participants were given the opportunity to ask questions before enrolment.

### Study site, participants, and sample collection

The study was carried out in Ibadan, the capital of Oyo state, located in the southwest of Nigeria. Pregnant women were recruited into the study in August 2018 from four hospitals: Our Lady of Apostle Catholic Hospital, Oluyoro; St. Mary General Hospital, Eleta; Adeoyo Maternity Teaching Hospital, Yemetu; and Beta Life Hospital, Agbowo. A total of 281 pregnant women gave consent to participate, in which 102, 108, 70 and one pregnant women were recruited from Oluyoro, St. Mary, Adeoyo and Beta Life, respectively. Participants were not tested for HIV, but their hospital antenatal records indicated that they were all HIV-negative. Age, gravidity, current participation in intermittent preventive treatment in pregnancy (IPTp), and other demographic information were obtained using a standardized questionnaire. A peripheral blood sample, 5 mL, was collected from each of the participants into labelled EDTA tubes. PCV was measured using the ruler method. The plasma samples obtained after centrifugation were stored at first at -20°C and then at -80°C until used.

### Parasitological examination

Thick and thin smears made directly from collected blood samples were stained with Giemsa. Parasitemia was calculated based on the number of parasites counted against 500 leukocytes and assuming 8000 leukocytes/µL of blood.

### Measurement of anti-PS by enzyme linked immunosorbent assay

Anti-PS IgG antibodies were measured using an ELISA kit (Orgentec, Germany) following the manufacturer’s instructions. Briefly, diluted samples (1:100), calibrators and controls were added to PS-precoated wells and incubated for 30 minutes at room temperature (RT). Plates were washed and HRP-labelled anti-human IgG was added to the wells and incubated for 15 min at RT. TMB (3,3’,5,5’-tetramethylbenzidine) was added and incubated for 15 min. The reaction was stopped by adding a stop solution and absorbance was read at 450 nm. Anti-PS antibody concentration was estimated from a standard curve.

### VAR2CSA production

The extracellular domain encompassing all six DBL domains (amino acid 1-2649) of the VAR2CSA (IT4VAR04) protein was expressed as a recombinant antigen with a C-terminal 8×His tag. In brief, a pcDNA3.4 plasmid encoding the protein was transfected into ExpiCHO-S™ cells (Thermo Fisher Scientific) using the Expifectamine™ CHO Reagent and the manufacturer’s max titer protocol. Culture supernatant was harvested seven days after transfection by centrifugation at 3500 × g for 15 minutes. The protein was purified from the supernatant by immobilized metal ion affinity chromatography (IMAC) using a HisTrap High-Performance column (GE-Healthcare) followed by size exclusion chromatography using a HiLoad 16/600 Superdex 200pg column (GE Healthcare). The purity and appropriate folding of the protein was verified by sodium dodecyl sulfate polyacrylamide gel electrophoresis (SDS-PAGE) under reducing and non-reducing conditions ( ± DTT) followed by Instant Blue (Expedeon) staining.

### Measurement of anti-VAR2CSA by ELISA

Anti-VAR2CSA IgG antibodies in the plasma samples were measured by standard ELISA. Maxisorb immunoplates were coated overnight at 4°C with 50 µL/well (1 µg/mL) of VAR2CSA in PBS. Plates were washed three times using PBS/0.05 Tween and then blocked with 10% skimmed milk/PBS/0.05% Tween 20 at RT for 2 hours. Washing was repeated and 50 µL/well of plasma samples (1:50) in 5% skimmed milk/PBS/0.05% Tween 20 were added in duplicates followed by incubation at RT for 1 h. Plates were washed again and 50 µL/well of HRP-conjugated goat anti-human IgG (Sigma) diluted 1:2000 in 5% skimmed milk/PBS/0.005% Tween 20 was added and incubated at RT for 1 h. After washing 50 µL/well of 2,2’-Azino-bis (3-ethylbenzothiazoline-6-sulfonic acid), ABTS, was added and color was allowed to develop for 30 min in the dark before absorbance was read at 405 nm.

### Haptoglobin measurement

Plasma haptoglobin levels were determined using an ELISA kit (BioSite) according to the manufacturer’s instructions.

### Data analysis

Data was analysed using Graphpad Prism version 9.0 (GraphPad Software, Inc., CA, USA) and STATA 12.1. Association between parameters was determined by Spearman’s correlation. Mann-Whitney tests were used to compare two groups and Kruskal-Wallis tests were used for comparing median PS and VAR2CSA antibodies between women of different gravidities. Association between PS antibody levels, age and gravidity was determined by multivariate regression analysis.

## Results

### General characteristics of the study population

A total of 281 pregnant women participated in this study and their age ranged from 18-43 years, median age was 30 years (**Table 1**). Gravidity ranged from 1-7 (2.2 ± 1.2) and was further categorized as primigravidae, secundigravidae and multigravidae (3-7 gravidities). Most of the participants, 96.4% (271), were permanent residents of Ibadan while 3.6% (10) visited Ibadan for their monthly antenatal visit from neighboring areas. Only 39.9% (112) of our study subjects participated in IPTp. The median gravidity between IPTp participants and non-participants was not significantly different (p=0.06). The adopted IPTp schedule was sulfadoxine/pyrimethamine as recommended by the WHO, starting in the first trimester. Almost half, 56.9% (160), of the pregnant women used insecticide treated nets.

**Table 1:** Baseline characteristics of participants.

Characteristics	n= 281
Age, years, median (range)	30 (18-43)
Gravidity	
Primigravidae, % (n) Secundigravidae, % (n) Multigravidae, % (n)	37 (103) 29 (81) 34 (97)
Bed net use *	
Yes, % (n) No, % (n)	57 (160) 43 (120)
Education	
Post-secondary, % (n) Secondary, % (n) Preprimary/primary, % (n)	66 (185) 32 (89) 2 (7)
IPTp	
Yes, % (n) No, % (n)	40 (169) 60 (112)
Residence	
Ibadan, % (n) Others, % (n)	96 (271) 4 (10)

*n = 280.

### Measurement of parasitemia, PCV and haptoglobin

The peripheral parasitemia ranged from 0-7856/μL (median, 128/μL) and the PCV ranged from 16.7-45.2% (median, 33.3%). 73.4% of the individuals were parasite positive. About a quarter of the women had PCV values below 30% and were therefore categorized as anemic. Haptoglobin levels ranged from 27 pg/mL to 4412 ng/mL.

### Anti-PS and anti-VAR2CSA IgG in anemic pregnant women

**Figure 1:**
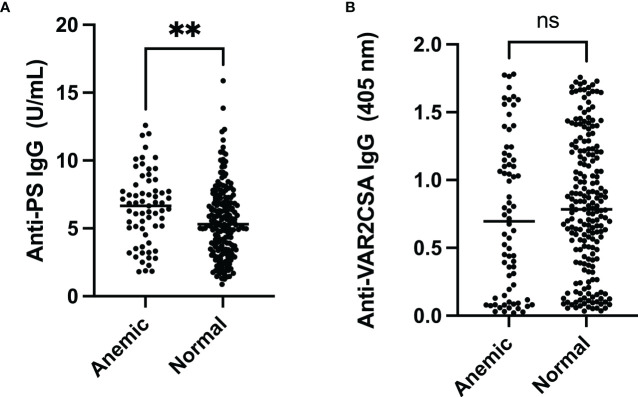
Comparison of anti-PS and anti-VAR2CSA antibody levels in pregnant women comparing anemic to those with normal PCV. **(A)** The median anti-PS antibody level was significantly higher in anemic pregnant women (** represent ≤ 0.01). **(B)** There was no difference in median anti-VAR2CSA antibody levels between anemic and normal pregnant women (p = 0.18). ns, not statistically significant.

Pregnant women were categorised based on their PCV values. The comparison of median PS- and VAR2CSA-specific antibodies showed that anti-PS antibody levels were significantly higher among the anemic women (p = 0.007) while anti-VAR2CSA antibodies did not differ between the two groups (p = 0.18) (**Figure 1**).

### Anti-PS-IgG in pregnant women by gravidity and parasitemia

**Figure 2:**
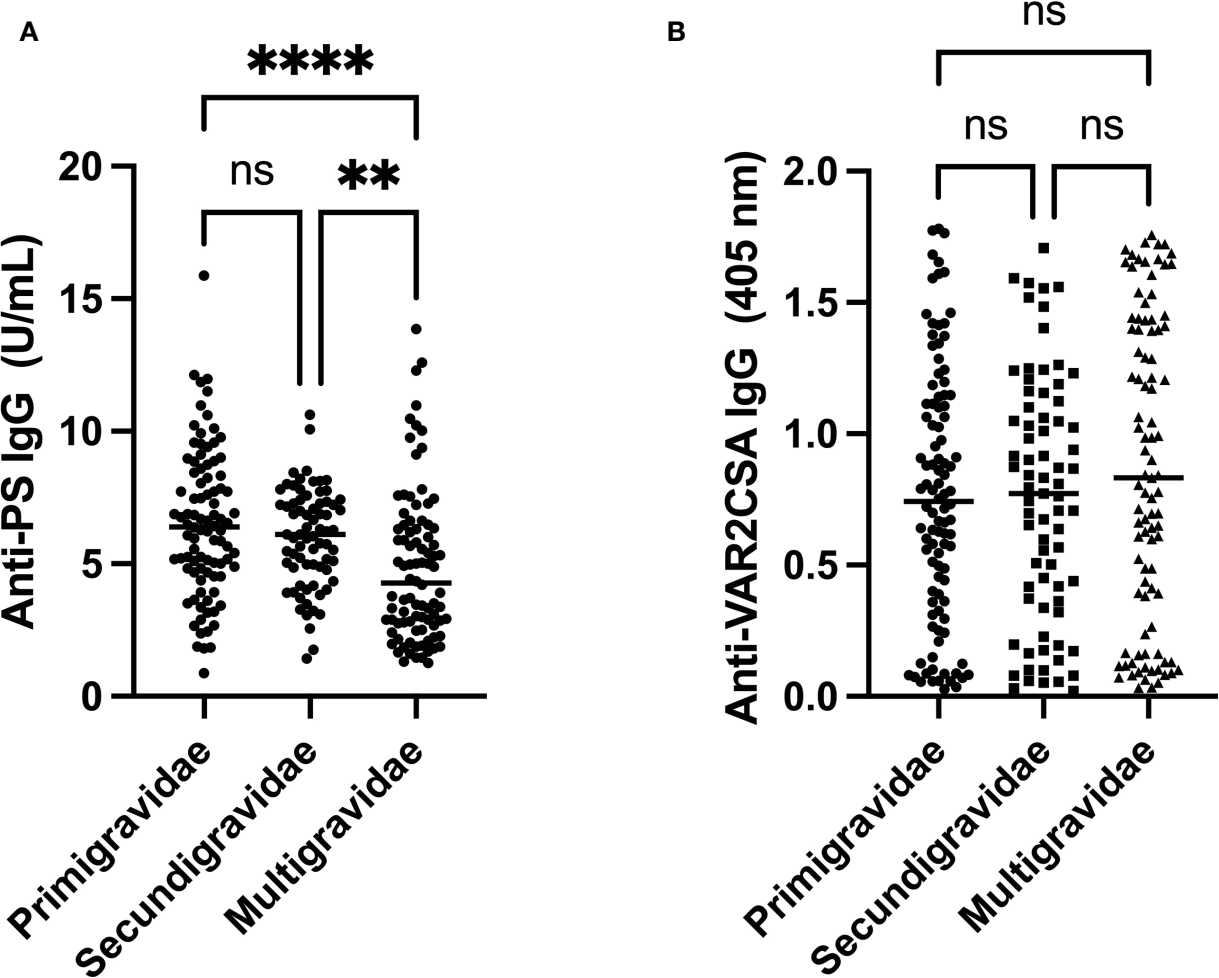
Comparison of anti-PS and anti-VAR2CSA antibody levels by gravidity. **(A)** Anti-PS levels were low in multigravidae when compared to both primigravidae and secundigravidae (** and **** represent p ≤ 0.01 and p < 0.0001, respectively). **(B)** Anti-VAR2CSA antibody seemed to be highest in multigravidae, but it was not statistically significant (p = 0.35). ns, not statistically significant.

Levels of PS-specific antibodies decreased with increasing parity (p<0.001) (**Figure 2A**). Furthermore, *post-hoc* analysis revealed no difference between median antibody levels between primigravidae and secundigravidae, but both primigravidae (p<0.001) and secundigravidae (p = 0.01) median antibody levels were significantly higher than that of multigravidae.

To determine whether the peripheral infection status of the pregnant women affected the pattern seen above, the women were further categorized into infected (positive blood smear) and non-infected (negative blood smear). There was no difference in median anti-PS antibody levels between infected and non-infected pregnant women (p=0.33). The multigravidae still had the lowest anti-PS antibody compared with primigravidae and secundigravidae in the infected (p=0.0003) and non-infected women (p=0.04).

### Anti-VAR2CSA IgG in pregnant women by gravidity

Antibodies against full length VAR2CSA was used as a measure of placental malaria exposure. Absorbances varied from 0.02 to 1.78. The top 25th and lower 75th percentiles of VAR2SCA antibody levels showed absorbances of 0.36 and 1.21, respectively. Multigravidae pregnant women had higher median VAR2CSA antibody than primigravidae and secundigravidae (**Figure 2B**), however this difference in median antibody level was not statistically significant (p = 0.35).

### Correlation between parameters

When PCV, parasitemia, age, haptoglobin, anti-PS and anti-VAR2CSA were correlated to each other using Spearman’s correlation, there was no correlation between anti-PS and anti-VAR2CSA antibody levels (r=-0.03, p=0.62) (**Table 2**). However, anti-PS antibodies correlated inversely with age (r =-0.16, p=0.01) and PCV (r =-0.14, p=0.02). No correlation was observed between anti-PS levels, parasitemia or haptoglobin levels. As expected, parasitemia correlated inversely with PCV (r = -0.15, p = 0.01).

**Table 2:** Correlation between some parameters.

	PCV	Parasitemia	Anti-VAR2SCA IgG Ab	Anti-PS IgG Ab	Haptoglobin level	Age
**PCV**		**-0,15(0,01)**	0,1(0,09)	**-0,14(0,02)**	-0,1(0,11)	**0,19(0,001)**
**Parasitemia**			-0,09(0,15)	0,05(0,42)	-0,04(0,58)	-0,04(0,52)
**Anti-VAR2CSA IgG Ab**				-0,03(0,62)	**0,21(0,001)**	0,06(0,35)
**Anti-PS IgG Ab**					0,07(0,28)	**-0,16(0,01)**
**Haptoglobin level**						-0,10(0,12)

Spearman’s correlation coefficients between different parameters are shown followed by p values in parentheses. Statistically significant correlation coefficients are shown in bold.

**Figure 3:**
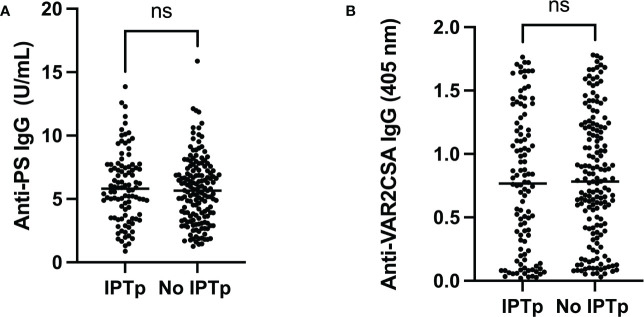
Comparison between pregnant women who participated (IPTp) or did not participate (No IPTp) in the IPTp program. There was no difference between the groups for levels of **(A)** Anti-PS (p=0,49) or **(B)** Anti-VAR2CSA (p=0,54). ns, not statistically significant.

When women, who participated in IPTp and those who did not, were compared (**Figure 3**), there was no difference between median levels of anti-PS antibodies (p = 0.49) or anti-VAR2CSA antibodies.

Since age also has an inverse relationship with PS antibody levels, we further tested the relationship between PS antibody level, age and gravidity in a multivariate regression analysis. Gravidity (β = 0.42) had significantly more influence on PS antibody levels than age (β = 0.1).

## Discussion

PS-specific antibodies appear in some autoimmune diseases and of viral, bacterial, and parasitic infections, including malaria (Skouri et al., 2008; Elkon and Silverman, 2012; Barber et al., 2019). We did not find any correlation between parasitemia and anti-PS levels, which is in agreement with an earlier study (Barber et al., 2019). It is furthermore consistent with a scenario where the measured PS-specific antibodies reflect previous rather than current parasitemia. Additionally, it has been shown that contact of CD11c^+^T-bet^+^ B cells, which are mainly responsible for PS production in mice and humans, with parasite DNA is sufficient to trigger anti-PS antibody production (Rivera-Correa et al., 2017; Rivera-Correa et al., 2019b).

Our observation that PS-specific antibody levels were elevated in anemic participants, and their correlation with PCV, is consistent with the proposed anemia-inducing effect of PS-specific antibodies through phagocytic clearance of opsonized RBCs exposing PS (Fernandez-Arias et al., 2016). Earlier studies have also consistently demonstrated inverse correlations between levels of PS-specific antibodies and hemoglobin (Barber et al., 2019; Rivera-Correa et al., 2019b). When there is anemia due to intravascular hemolysis of RBCs, haptoglobin levels are expected to fall. Haptoglobin is a plasma glycoprotein that binds free hemoglobin to eliminate its toxic effects However, we did not observe any relationship between PS antibodies and haptoglobin plasma levels. Since haptoglobin is also an acute-phase protein that can rise due to presence of any infection or inflammation, and none of the participants in our study had clinical symptoms of malaria, it is possible that levels of haptoglobin measured by us were being controlled by other factors/infections that were not related to malaria parasite schizogony. The lack of correlation between anti-PS antibody and haptoglobin levels may be due to the ability of anti-PS antibodies to cause anemia through phagocytosis by macrophages as shown in mice (Fernandez-Arias et al., 2016) and not only by hemolysis. The level haptoglobin found in our study was very low compared to the international reference range of 300-2150 μg/mL(Dati et al., 1996). This is not surprising because an earlier study had demonstrated a lower normal range of 25-92 μg/mL in a Nigerian population, even though different from our study population (Eluke et al., 2018). Haptoglobin plasma levels have been shown to be influenced by genotype (Teye et al., 2004; Fowkes et al., 2006; Abah et al., 2018), and by geographical location and age (Fowkes et al., 2006). The possible effect of genetics on haptoglobin levels is particularly important here, because one of the earliest studies on haptoglobin population genetics found that more than 30% of a Yoruba population in Nigeria lacked haptoglobin expression (Allison et al., 1958), a condition called anhaptoglobinemia. The majority of participants in this current study belonged to the Yoruba ethnic group. Furthermore, haptoglobin levels have been found to fluctuate according to gestational stage (Haram et al., 1983); so haptoglobin levels may be modulated by pregnancy-associated factors that complicate its relationship with anti-PS antibody levels, if there is any. Generally, results from studies on the relationship between haptoglobin levels and intravascular hemolysis during malaria are inconclusive (Rogerson, 2006), and our study confirms that it is difficult to use haptoglobin as a measure of intravascular hemolysis in this endemic area.

Artemisinin is used as part of IPTp in our study area, and its use has been linked to PS exposure on RBC (Alzoubi et al., 2014). However, no difference was observed in anti-PS antibody levels between pregnant women who participated in IPTp and those who did not. Self-medication for malaria is a relatively common practice in Nigeria (Omolase et al., 2007; Wegbom et al., 2021), therefore women who were not formally participating in IPTp could have been using artemisinin, other anti-malarials or herbal extracts.

Our study found no correlation between antibodies produced against full-length VAR2CSA and anti-PS. We speculate that these antibodies are from different B cell sources. Indeed, PS antibodies have been shown to be produced only by atypical T-bet^+^ FcRL5^+^ memory B cells, and no relationship were found between anti-PS antibodies and other B cell subsets (Rivera-Correa et al., 2019b). The possibility that VAR2CSA and anti-PS antibodies arise through different mechanistic pathways may make it easy to downregulate PS antibodies and its associated pathologic effects without affecting the useful protective functions of VAR2CSA during pregnancy. Similar levels of VAR2CSA IgG response were observed among the three gravidity groups used in our study; this indicates that there is a high level of exposure to VAR2CSA in our study population such that multigravidae had only a slightly higher antibody response, which was not statistically significant, compared to responses seen in primigravidae and secundigravidae. A high level of exposure to malaria parasites is expected because Ibadan, our study area, is endemic for malaria. A study in a Cameroonian population have also found antibody responses against full-length VAR2CSA to be similar in pregnant women of different gravidity (Vanda et al., 2021), but placental malaria was common in all of them. Importantly, we found that multigravidae had the lowest anti-PS antibody level when compared with secundigravidae and primigravidae. Reduced levels of anti-PS antibodies have earlier been demonstrated in multigravidae without active placental infection, but not in those with active or history of placental malaria (Owens et al., 2005). Placental infection was not determined in the current study, but multigravidae still had the lowest anti-PS antibody levels irrespective of the presence or absence of peripheral malaria parasites. Although the etiology of anemia during pregnancy in malaria endemic regions is multifactorial, it is likely that low levels of anti-PS antibodies may contribute to a better hematological profile and less severe consequences of malaria seen in multigravidae (Verhoeff et al., 1999; Guyatt and Snow, 2001). We could not find any studies of transplacental transfer of anti-PS antibodies in the context of malaria, but it is not uncommon for transplacentally transferred autoantibodies to impact fetuses or neonate health (Giacoia, 1992; Vanoni et al., 2017). This research field obviously requires more investigations in multiple malaria endemic regions as elevated levels of anti-PS antibody could also have some anti-malarial activities in multigravidae with active or history of placental malaria (Owens et al., 2005).

In conclusion, PS-specific antibodies correlated with anemia measured by PCV but were not related to VAR2CSA-specific antibodies in pregnant women. Less severe pathological consequences of malaria generally observed in multigravidae may not be due to protective functions of VAR2CSA-specific antibodies alone, but also by the reduction in the pathologic effects of anti-PS antibodies. More work is needed in other endemic areas to gain more insights into the functions of anti-PS antibodies and other malaria-induced autoimmune antibodies during pregnancy.

## Data availability statement

The original contributions presented in the study are included in the article/supplementary material. Further inquiries can be directed to the corresponding authors.

## Author contributions

Conceptualization and design: MT and KP. Sample collection: AF, NM, and MT. Laboratory work and results analysis/interpretation: AF, NM, MT, BS, AT, MQ, LH and KP. Writing and revision of manuscript: AF, NM, MT, BS, AT, MQ, LH and KP. All authors contributed to the article and approved the submitted version.
